# Room temperature spin valve effect in NiFe/WS_2_/Co junctions

**DOI:** 10.1038/srep21038

**Published:** 2016-02-12

**Authors:** Muhammad Zahir Iqbal, Muhammad Waqas Iqbal, Salma Siddique, Muhammad Farooq Khan, Shahid Mahmood Ramay

**Affiliations:** 1 Faculty of Engineering Sciences, GIK Institute of Engineering Sciences and Technology, Topi 23640, Khyber Pakhtunkhwa, Pakistan; 2 Department of Physics & Astronomy, Georgia State University, Atlanta, GA 30303, USA; 3 Department of Physics, College of Science, Majmaah University, Al-Zulfi 11932, Saudi Arabia; 4 Department of Bioscience & Biotechnology, Sejong University, Seoul 143-747, Korea; 5 Department of Physics & Graphene Research Institute, Sejong University, Seoul 143-747, Korea; 6 Physics & Astronomy Department, College of Science, King Saud University, Riyadh 11451, Saudi Arabia

## Abstract

The two-dimensional (2D) layered electronic materials of transition metal dichalcogenides (TMDCs) have been recently proposed as an emerging canddiate for spintronic applications. Here, we report the exfoliated single layer WS_2_-intelayer based spin valve effect in NiFe/WS_2_/Co junction from room temperature to 4.2 K. The ratio of relative magnetoresistance in spin valve effect increases from 0.18% at room temperature to 0.47% at 4.2 K. We observed that the junction resistance decreases monotonically as temperature is lowered. These results revealed that semiconducting WS_2_ thin film works as a metallic conducting interlayer between NiFe and Co electrodes.

Two-dimensional (2D) nanomaterials have been already established to have prodigious potential for application in the field of spintronics^1^,^2^,^3^,^4^,^5^,^6^,^7^,^8^,^9^. The 2D transition-metal dichalcogenides (TMDCs) have attractive properties such as bandgap, atomically thin layered structure and a promising material for active channel in field-effect transistor applications. In particular, tungsten- and molybdenum-based TMDC are attracting materials due to semiconducting and optoelectronics properties^10^,^11^,^12^. The tungsten disulfide (WS_2_) has a fascinating property that it shows direct bandgap for monolayer while have indirect bandgap for bulk. The bulk WS_2_ have an indirect bandgap (1.4 eV) but goes to a direct bandgap (2.1 eV) material when exfoliated into the monolayer^13^. The structure of single layer of WS_2_ crystals is formed by covalently bonded in-plane S-W-S atoms, which contain of two sheets of S atoms and one sheet of W atoms and are hexagonally packed. In general wide band gap oxides such as Al_2_O_3_^14^,^15^,^16^ or MgO are previously being utilized as a nonmagnetic spacer in spin valve devices^17^,^18^,^19^,^20^,^21^. The basic principal of spin valve comprises of two ferromagnetic metal layers decoupled by a non-magnetic insertion, which permits parallel and antiparallel alignment of the magnetizations of two magnetic layers. The magnetoresistance of a spin valve can be determined from the magnetization alignment configuration between two ferromagnetic electrodes and controlled by the external magnetic field. Recently, there has been extensive interest in the spin-dependent properties of 2D materials such as: graphene, hexagonal boron nitride, molybdenum disulfide and it became more incorporative platform for non-magnetic interlayer spacer between two ferromagnetic electrodes in current perpendicular to plane spin valve device structures^1^,^6^,^7^. There have been efforts to explore a variety of new spin valve structures of 2D materials with single interlayer spacer to few layers^1^,^2^,^3^,^4^,^5^,^6^,^7^,^8^,^9^, and further continue to be fully investigated for new materials.

Here, we report on the first fabrication and characterization of tungsten disulfide based spin valve effect in the junction comprising top electrode (Co) and bottom electrode of Permalloy (Py, Ni_81_Fe_19_) film and WS_2_ as an interlayer. The magnetoresistance show two resistance states depending on the magnetization alignment configuration between two electrodes. The spin valve signals are observed from room temperature to 4.2 K and having magnetoresistance ratios of 0.18% at 300 K to 0.47% at 4.2 K. We have also studied the basic functionality of semiconducting WS_2_ film junction resistance as function of temperature.

## Results and Discussion

### Characterization of WS_2_ spin valve

**Figure 1:**
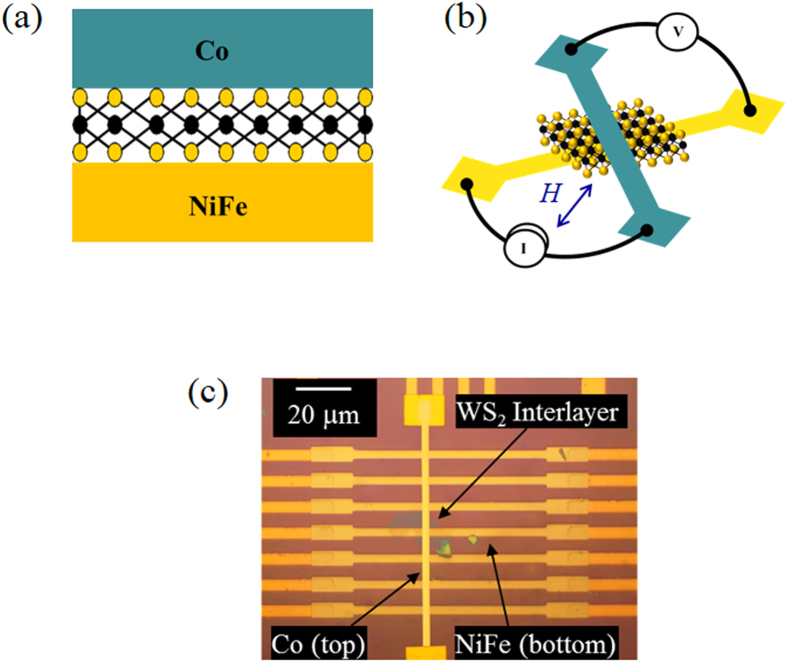
(**a**) Schematic view of WS_2_ spin valve consisting of top Co electrode, bottom NiFe electrode and a WS_2_ interlayer. (**b**) The measurement configuration of spin valve device. Magnetic field (*H*) is applied in plane and at oblique angle to the ferromagnetic electrode axis. (**c**) Optical micrograph of the complete NiFe/WS_2_/Co device.

Figure 1(a) shows a schematic of WS_2_ spin valve consisting of top Co electrode, bottom NiFe electrode and a WS_2_ interlayer. The device structure and the measurement configuration are shown in Fig. 1(b). While current flows from Co to NiFe through WS_2_ interface, voltage is measured between Co and NiFe. The in-plane magnetic field *(H)* was applied at 45° to the direction of the ferromagnetic (FM) electrodes. Therefore the magnetization alignments can be made semi-parallel or semi-antiparallel by sweeping *H*. The optical micrograph of the complete device is portrayed in Fig. 1(c).

**Figure 2:**
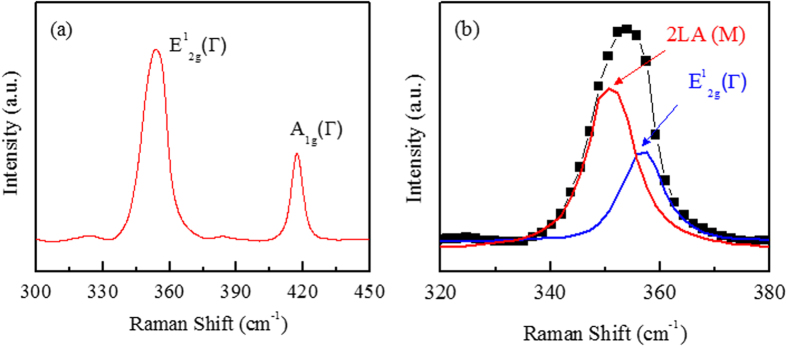
(**a**) Raman spectra (514 nm wavelength) of single layer WS_2_ film after transferred to the Si/SiO_2_ substrate. (**b**) Lorentzian peaks fitting to identify the positions 2LA(M) and first-order 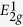
mode.

Figure 2(a) shows Raman spectrum measured for the WS_2_ film. Raman spectra of the SL-WS_2_, film show strong signals of in-plane 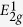, out-of-plane 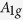, and vibration second-order 2LA(M) modes. The first-order 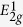
and 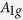 optical modes were used to identify the 2D materials, such as MoS_2_, but the intensity of the 2LA(M) mode at 352 cm^−1^ was considerably higher for WS_2_. The Raman peak positions of 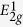
and 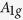
for SL-WS_2_ are 355.2 and 417.7 cm^−1^ respectively. The frequency difference between Raman 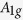 and 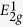 modes 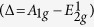 is about 62.5 cm^−1^, which indicates a single layer of the WS_2_ film^11^,^22^,^23^. However, the 2LA(M) mode was overlapped with the first-order 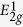
mode at 355.2 cm^−1^, Lorentzian peaks fitting clarify the contribution of each peak as shown in Fig. 2(b)11.

### Spin valve effect and current-voltage characteristics in WS_2_-interlayer junction

**Figure 3:**
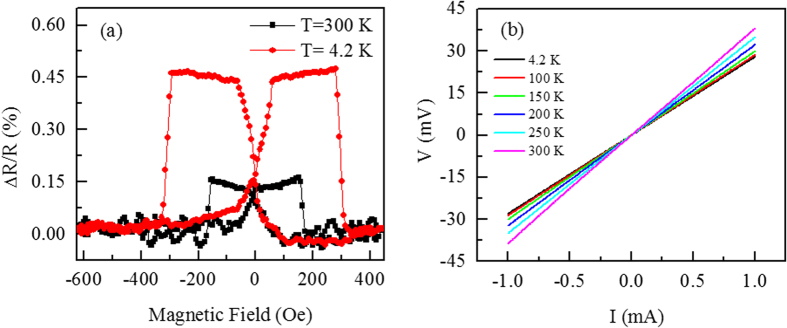
(**a**) Magnetoresistance ratio of the NiFe/WS_2_/Co spin valve device as a function of magnetic field (*H*) at 4.2 K and 300 K. Magnetoresistance ratio is in the high (low) state for the antiparallel (parallel) magnetization configuration between NiFe and Co. (**b**) The current-voltage (*I*–*V*) characteristics of the NiFe/SL-WS_2_/Co junction at various temperatures range from 4.2 K to 300 K.

The spin valve effect was studied by examining the relative magnetoresistance ratio (MR), which is defined by MR = [R_AP_ − R_P_]/R_P_. Here, R_AP_ is the magnetic field dependent resistance and R_P_ is the resistance corresponding to the parallel alignment of magnetizations. Figure 3 (a) shows MR signal of NiFe/SL-WS_2_/Co spin valve device. The value of MR shows bistable states where high (low) resistance appears in the antiparallel (parallel) magnetization configuration between Co and NiFe. The magnitude of MR value is observed of the order of 0.18% at 300 K and 0.47% at 4.2 K. We have further investigated the nature of NiFe/SL-WS_2_/Co junction by measuring current-voltage (*I*–*V*) characteristics. The *I*–*V* curves of the junctions at various temperatures range from 4.2 K to 300 K are shown in Fig. 3(b). The linear behavior of *I*–*V* curves indicate the ohmic characteristics of the junctions.

### Temperature dependence of the spin valve effect

**Figure 4:**
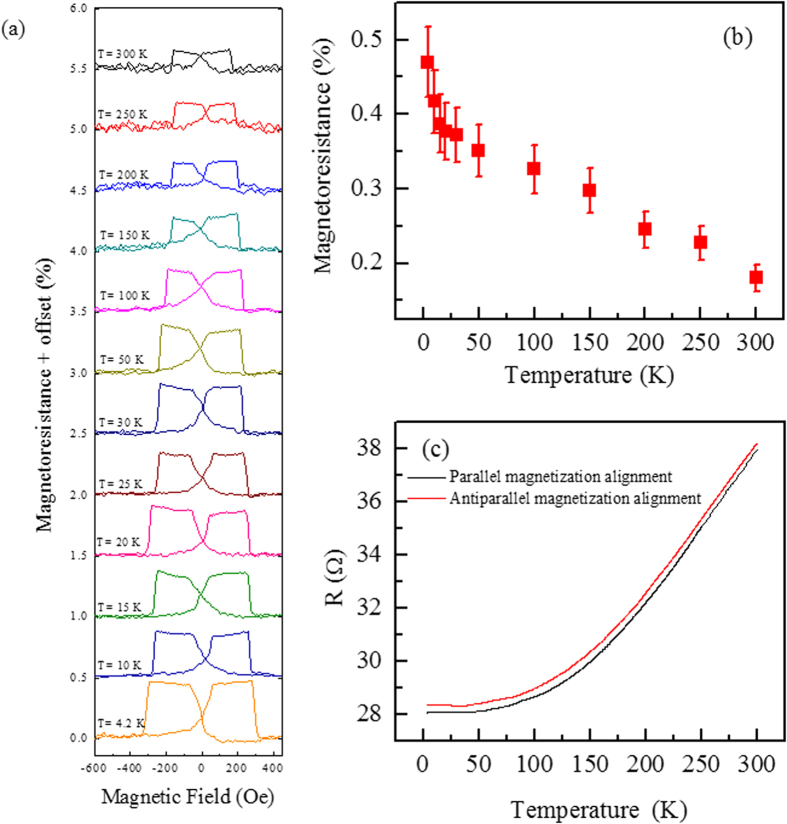
(**a**) Magnetoresistance ratio (MR) as a function of magnetic field (*H*) for the WS_2_ spin valve at various temperatures. The spin valve signals are observed at all temperatures in the experiment. However, the magnitude of MR increases by reducing the temperature. (**b**) The variation in the MR values as a function of temperature. (**c**) The junction resistance WS_2_ spin valve as a function of temperature.

The WS_2_-interlayer spin valve device reveals a sequence of MR curves at various temperatures ranging from 300 K to 4.2 K as shown in Fig. 4(a). The magnitude of the spin valve signal having MR monotonically decreases as the temperature is increased. The decrease of MR at higher temperature can be attributed to many possible origins such as magnetic impurity scattering, surface states, and thermal smearing of the electron energy distribution in ferromagnetic^24^. The similar temperature dependence has been observed in magnetic tunnel junction^25^. The switching points of MR curves becomes broader at low temperature due to increasing switching fields of FM as thermal agitation is weakened. The trend of increment in the MR values as a function of temperature is shown in Fig. 4(b).

The resistance of WS_2_ interlayer junction in the parallel and antiparallel magnetization alignment is measured as a function of temperature. The junction resistance decreases monotonically as temperature is lowered as shown in Fig. 4(c). These observations suggest that the role of WS_2_ film as metallic interlayer instead of semiconducting nature. The theoretical evidence also suggests that the metallic nature of metal dichalcogenide films^7^. The distance between the ferromagnetic surface atoms and nearest S atoms is d = 2.1 Å. Therefore, the bonding between metal dichalcogenide film and ferromagnetic electrode permits a strong wave-function overlap between the Mo/W and ferromagnetic states. The junction shows metallic nature with the pristine gap of metal dichalcogenide film being absent leading to a large transmission. Because the Fermi level is pinned at the conduction band minimum and therefore it is also evinced that the projected densities of states on Mo/W atoms shows no energy gap.

### Current bias dependence of the spin valve effect

**Figure 5:**
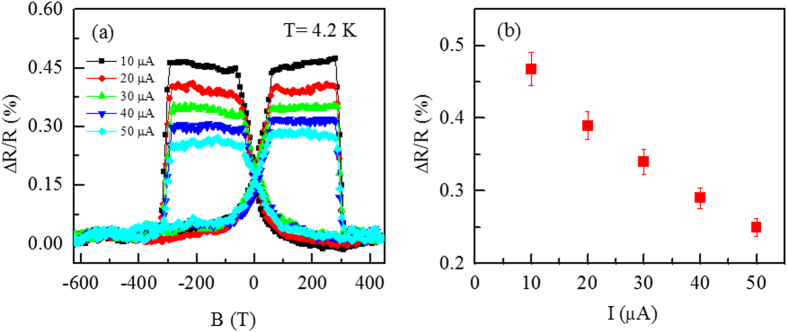
(**a**) The magnetoresistance of spin valve as a function of magnetic field at different bias current values range from 10 μA to 50 μA. (**b**) The change in magnetoresistance for different bias current values.

The magnetoresistance variation is examined by applying various bias current on the junction. Figure 5 (a) shows the spin valve effect at different bias currents from 10 μA to 50 μA. The results reveal that by increasing bias current the magnetoresistance is decreased. The trend of change in magnetoresistance as a function of bias current is shown in Fig. 5(b). The decrease of magnetoresistance by increasing bias current is attributed to the localized trap states in the interlayer^26^.

## Conclusions

In conclusion, we have demonstrated spin valve effect in single layer WS_2_ film as an intervened layer between NiFe and Co electrodes. Our results show that the spin valve effect of WS_2_ junction have relative magnetoresistance in spin valve effect increases from 0.18% at room temperature to 0.47% at 4.2 K. We observed that junction resistance decreases monotonically by reducing the temperature. These results revealed that semiconducting WS_2_ thin film works as a metallic conducting interlayer between NiFe and Co electrodes. The WS_2_ interlayer spin valve junction is expected to be an attractive candidate for future spintronic devices and may contribute to open new avenues for the industrial application.

## Experimental Section

### Material and device fabrication

The single layer WS_2_ film was obtained by mechanically exfoliating natural WS_2_ using adhesive tape and then the flake was transferred onto the pre-patterned Py electrodes on SiO_2_/Si substrate using wet transfer method. The single layer (SL) was initially identified by optical microscopy and then further confirmed by Raman spectroscopy. The device fabrication is categorized into three different parts; bottom Py film, WS_2_ film transfer, and top Co film. Before that the outer patterns were made on Si substrate with SiO_2_ thickness of 300 nm using photo-lithography and Cr/Au (5/30 nm) deposition. The bottom Py electrodes were initially patterned using e-beam lithography. A standard lift-off procedure was used to obtain the bottom electrode lines after evaporating Py with thickness of 55 nm. Then a single layer WS_2_ was transferred on top of bottom Py electrodes. In the subsequent process top Co electrode with thickness of 75 nm was patterned by using e-beam lithography and lift-off process. The width of both top and bottom electrodes were 3 μm.

### Device characterization and measurement setup

Renishaw Raman micro-spectrometer was use to characterize the WS_2_ samples. The laser wavelength of the spectrometer was 514 nm, and the power was kept below 1.0 mW to avoid laser-induced heating. The transport measurements for WS_2_ based spin valve devices were performed using ac lock-in technique. The bias currents was kept 10 μA for temperature dependent spin transport measurements and further increased up to 50 μA to study the effect of bias dependence. The devices were cooled by liquid helium for low temperature measurements and the temperature was modulated by Lake Shore 331 temperature controller.

## Additional Information

**How to cite this article**: Iqbal, M. Z. *et al.* Room temperature spin valve effect in NiFe/WS_2_/Co junctions. *Sci. Rep.* **6**, 21038; doi: 10.1038/srep21038 (2016).
